# Comparison of Serum Zinc and Copper levels in Children and adolescents with Intractable and Controlled Epilepsy

**Published:** 2014

**Authors:** Zeynab KHERADMAND, Bahram YARALI, Ahad ZARE, Zahra POURPAK, Sedigheh SHAMS, Mahmoud Reza ASHRAFI

**Affiliations:** 1 Children’s Medical Center, Pediatric Center of Excellence, Tehran University of Medical Sciences, Tehran, Iran; 2 Immunology, Asthma and Allergy Research Institute, Children’s Medical Center, Tehran University of Medical Sciences, Tehran, Iran; 3 Department of Pediatric Neurology, Children’s Medical Center, Pediatric Center of Excellence, Tehran University of Medical Sciences, Tehran, Iran

**Keywords:** Serum copper, Serum zinc, Intractable epilepsy, Controlled epilepsy

## Abstract

**Objective:**

Trace elements such as zinc and copper have physiological effects on neuronal excitability that may play a role in the etiology of intractable epilepsy. This topic has been rarely discussed in Iranian epileptic patients.

This study with the analysis of serum zinc and copper levels of children and adolescents with intractable and controlled epilepsy may identifies the potential role of these two trace elements in the development of epilepsy and intractability to antiepileptic drug treatment.

**Materials & Methods:**

Seventy patients between the ages of 6 months to 15 years that referred to Children’s Medical Center with the diagnosis of epilepsy, either controlled or intractable to treatment enrolled in the study. After informed parental consent the levels of serum zinc and copper were measured with atomic absorption spectrophotometer and analyzed with SPSS version 11.

**Results:**

35 patients were enrolled in each group of intractable (IE) and controlled epilepsy (CE). 71.45% of the IE and 25.72% of the CE group had zinc deficiency that was statistically significant. 48.58% of the IE and 45.72 of the CE group were copper deficient, which was not statistically significant.

**Conclusion:**

Our findings showed significant low serum zinc levels of patients with intractable epilepsy in comparison with controlled epilepsy group.

We recommend that serum zinc level may play a role in the etiology of epilepsy and intractable epilepsy therefore its measurement and prescription may be regarded in the treatment of intractable epilepsy.

## Introduction

Seizure disorder is one of the most common neurological diseases in children and occurs at least one time in 4-10% of children in the first 16 years of life (1).

Occurrence of seizure in children is not only a terrible experience for the parents but also have many neurologic, cognitive, psychological, and social complications for the child and adolescent.. Recurrent unprovoked seizures called epilepsy and its diagnosis are done when two or more unprovoked seizures have occurred at intervals longer than 24 hours intervals (1). The annual prevalence of epilepsy is 0.5-1% and its lifetime cumulative incidence is 3% (1). Epilepsy begins in childhood in more than half of the cases. Approximately 50 million people in the world have epilepsy, up to one -third of whom continue to have seizures despite appropriate drug treatment (2). 

Despite the importance of identifying refractory epilepsy, due to its comorbidities and sometimes mortalities different definitions presented in the literature. Drug resistant epilepsy may be defined as failure of adequate trials of two tolerated and appropriately chosen and used antiepileptic drugs (AEDs) schedules (whether as monotherapies or in combination) to achieve sustained seizure freedom. (3). In addition to number of AEDs consumption, the frequency of seizures and duration of follow-up are important. Berg and Shinnar recommended an average of more than one seizure per month for 18 months and no more than 3 consecutive month’s seizure free during the follow-up period (4, 5). In our previous study that was conducted for the role of antioxidants in intractable epilepsy (2007), the patients categorized as intractable when they developed at least 1 seizure in a 6-month period despite being treated with at least 2 antiepileptic drugs(6).

Oxidative radicals that are produced during the metabolism of the brain, result in neuronal membrane damage, instability and epileptic discharges. 

Detoxification of the oxidative radicals is a major role of antioxidant enzymes such as glutathione peroxidase (GPx) which is a selenium-dependent enzyme and superoxide dismutase (SOD) that zinc and copper are its major constituents (7).

Current study was carried out to identify the role of the trace elements in the etiology of epilepsy and its intractability to treatment.

## Materials & Methods

This cross-sectional study was conducted on children and adolescents referred to Children’s Medical Center during March 2011-April 2012 with the diagnosis of epilepsy. Epileptic patients between the ages of 6 months to 15 years divided in two groups of intractable and controlled with AEDs. Symptomatic epilepsy due to Central Nervous System infections or metabolic and neurodegenerative disorders were excluded from the study group.

Other exclusion criteria were clinical symptoms of zinc and copper deficiency and anemia. 

At least one seizure in a 6-month period despite being treated with at least two antiepileptic drugs was used for the inclusion of our patients. This definition seems more practical than Berg and Shinnar definition. At least one year consumption of AEDs was necessary criteria for inclusion of the patients.

The sample size was designed according to previous studies and epidemiologist consults, that were 35 patients in each group. Necessary data completed in designed questioners such as sex, gender, perinatal history, family history of epilepsy or febrile seizure and consumption of supplements (iron and zinc). After informed consent of parents, 35 patients with inclusion criteria of the study recruited in each group. Six 6 ml blood samples were collected from subjects with 1 ml of EDTA and its serum separated. Serum samples were stored frozen at -70°C until measurement of serum zinc and copper levels. The samples sent to the Research Center of Immunology, Asthma and Allergy. Cell Blood Counts (CBC) analysis were performed by SYSMEX k21 cell counter (medical electronics, TOA, Kobe, Japan) and zinc and copper measurements were done by atomic absorption spectrophotometer (GBC, SENSAA, Australia). The Normal range of zinc was considered as 60-90 μg/dl between the ages of 1-12 months, 80- 110 μg/dl between the ages of 1-10 years, 90-120 μg/dl between the ages of 10-15 years, and the normal range of copper was considered as 40-80 μg/dl in all age groups (8,9,10). Data were analyzed with SPSS (version 11) and variables were analyzed with T-test and Chi-Square. 

The P value was considered significant below 0.05. 

## Results

Seventy patients enrolled that consisted of 40 males and 30 females, between the ages of 6 months to 15 years.

Overall 70% of the patients were treated with conventional antiepileptic drugs and 30% of them were treated simultaneously with both conventional and new AEDs. 62.9% of the patients had perinatal insults and in 37.1% of theme perinatal period was unremarkable. Family histories of epilepsy were seen in 31.4% of cases and family history of febrile seizures were seen in 27.1% of the patients. Seizure and epilepsy type classification of patients were as generalized tonic-clonic (70%), complex partial seizure (22.9%), infantile spasm (5.7%) and simple partial seizure (1.4%). 

**Fig 1:**
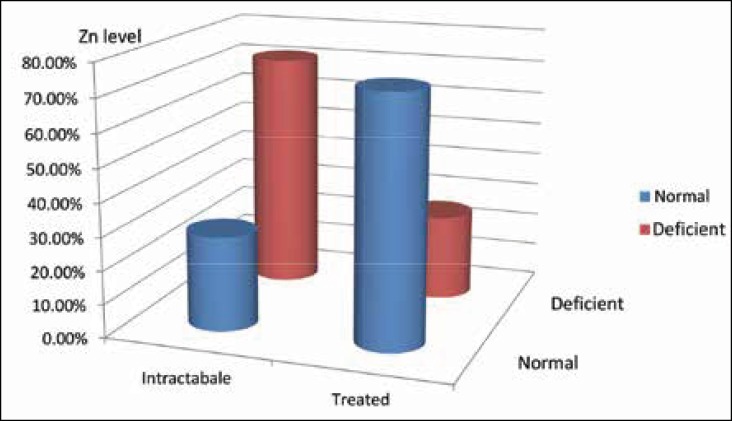
Serum zinc levels in patients with intractable epilepsy and epilepsy responding to treatment

Twenty-five patients (71.45%) in the intractable epilepsy group had zinc deficiency and 10 patients (28.55%) had normal zinc levels. Nine patients (25.72%) in the controlled epilepsy group had zinc deficiency and 26 patients (74.28%) had normal zinc levels (Figure1). 

Patients with the intractable epilepsy had significantly decreased levels of serum zinc in comparison with the controlled group (P value <0.001). 

**Fig 2:**
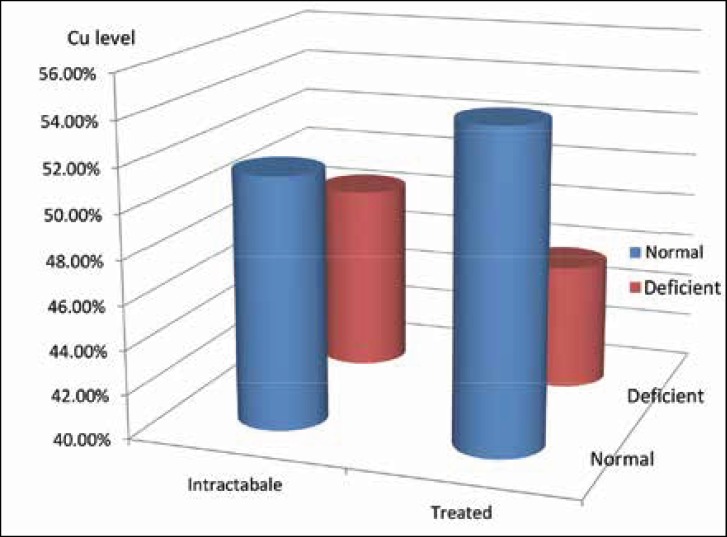
Serum copper levels in patients with intractable epilepsy and epilepsy responding to treatment

Eighteen patients (51.42%) in the intractable epilepsy group had normal serum copper level and 17 patients (48.58%) were copper deficient. Nineteen patients (54.28%) in the controlled epilepsy group had normal serum copper level and 16 patients (45.72) patients were copper deficient (Figure 2).

There was no statistically significant difference between serum copper levels of intractable and Controlled epilepsy group (P value =0.811).

**Fig 3:**
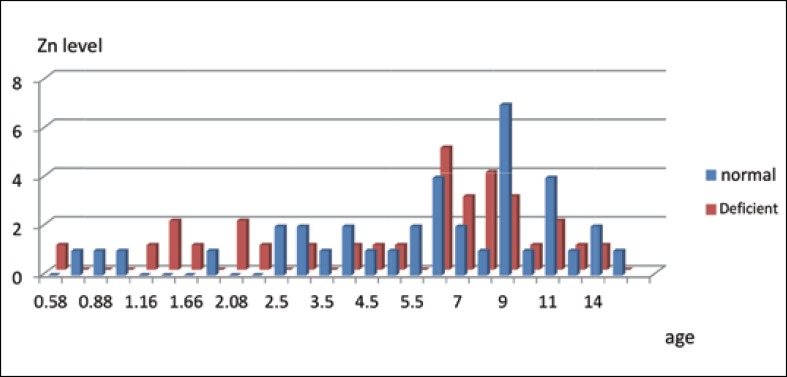
Serum zinc levels of patients according to age range

**Fig 4:**
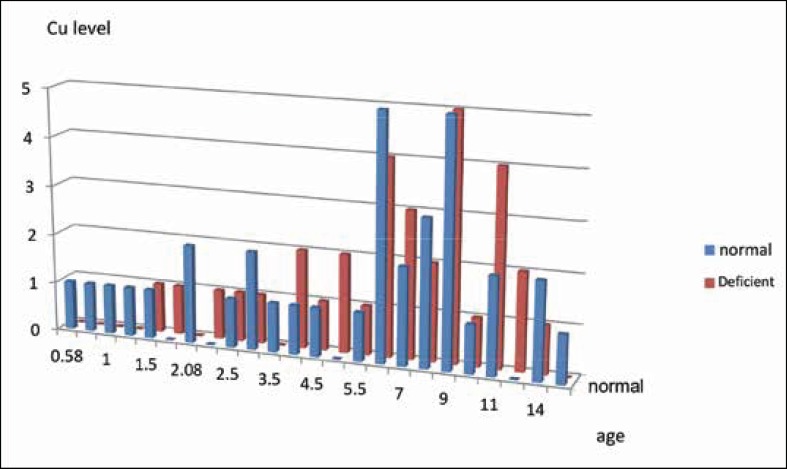
Serum copper levels of patients according to age range

According to the analysis, the highest numbers of cases in the zinc deficient group were between the ages of 6-8-years (Figure 3) and the highest numbers of cases in the copper deficient group were between the ages of 6-9 years (Figure 4).

## Discussion

About 70% of epileptic patients will be controlled with AEDs, but 20-25% of the patients do not have significant improvement in the seizure control even with the consumption of 2-3 AEDs (1, 11, 12). Epilepsy occur during childhood in more than 50% of cases, therefore refractory epilepsy is a major problem in this age group. 

Diagnostic confirmation of intractable epilepsy needs exclusion of epilepsy mimickers, incorrect drug selection or combination and unidentified underlying disorders (1).

Zinc is the second most abundant trace element in the body and is a cofactor for almost 300 enzymes (8,9). Copper is the third most abundant trace element in the body and is involved in electron transport and oxidation reactions (8,9). This shows the crucial role of zinc and copper in such reactions. The reasons for pharmacoresistancy of some types of epilepsy are not clear. Several studies published about the role antioxidants and some trace elements in the pathogenesis of seizure disorder (6,13,14, 15,16,17).

Glutaric acid decarboxylase enzyme activity is regulated by zinc. This enzyme plays a critical role in the synthesis of gamma-aminobutyric acid (GABA), which is a major neuron inhibitor (15). Previous studies have shown that levels of GABA are reduced in the Cerebrospinal fluid (CSF) of children with seizure disorders (15). Many ionic channels such as sodium and T-Type channels and GABA receptors which are activated by zinc and copper affect specific forms of epilepsy. Although specifying the impact of these ions as stimulants or inhibitory is not easily possible without great effort (15, 16). 

Experimental observations showed that zinc have both stimulatory and inhibitory effect on seizure activities. 

During epileptic seizure activities of humans and animal models, the distribution of micronutrients such as zinc and copper affected in the brain and peripheral tissues. Apparently epileptic seizure activity in mice has been reduced by giving copper and increased by zinc deprivation (15).

In the Wojciak et al study significant lower serum zinc levels in patients with epilepsy were seen, in comparison with control healthy group. In this study serum copper level of epileptic patients were higher than healthy control group (12). In the Seven et al study patients with idiopathic intractable epilepsy had significantly decreased levels of serum zinc in comparison with healthy children (17). In our study patients with the intractable epilepsy had significantly decreased levels of serum zinc in comparison with the controlled group ((P value <0.001). This finding is similar to previous studies of Wojciak et al and Seven et al, but the novelty of this study was the comparison of serum zinc between the intractable and controlled epilepsy group. This may confirm the role of zinc in the pathogenesis of unresponsiveness or resistance to the AEDs effects. In our study there was no statistically significant difference between serum copper levels of intractable and controlled epilepsy group (P value =0.811), although 48.58% of the intractable epilepsy group and 45.72 of the controlled epilepsy group were copper deficient. In the previous study of Wojciak et al serum copper level of epileptic patients were higher than healthy group and they recommended that epilepsy may increase the copper level, but our study did not confirmed this hypothesis. It seems that more studies must be designed about the role of copper in the pathogenesis of epilepsy.

**In conclusion, **we recommend that serum analysis of trace elements especially zinc must be considered in the workup of epilepsy and intractable epilepsy and prescription of zinc supplements may be useful in the prevention and treatment of epilepsy.
